# The crimson conundrum: heme toxicity and tolerance in GAS

**DOI:** 10.3389/fcimb.2014.00159

**Published:** 2014-11-05

**Authors:** Ankita J. Sachla, Yoann Le Breton, Fahmina Akhter, Kevin S. McIver, Zehava Eichenbaum

**Affiliations:** ^1^Department of Biology, College of Arts and Sciences, Georgia State UniversityAtlanta, GA, USA; ^2^Department of Cell Biology and Molecular Genetics and Maryland Pathogen Research Institute, University of MarylandCollege Park, MD, USA

**Keywords:** redox sensing, oxidative damage, heme adaptation, Gram-positive, PefRCD, chaperones, transcriptome analysis

## Abstract

The massive erythrocyte lysis caused by the Group A Streptococcus (GAS) suggests that the β-hemolytic pathogen is likely to encounter free heme during the course of infection. In this study, we investigated GAS mechanisms for heme sensing and tolerance. We compared the minimal inhibitory concentration of heme among several isolates and established that excess heme is bacteriostatic and exposure to sub-lethal concentrations of heme resulted in noticeable damage to membrane lipids and proteins. Pre-exposure of the bacteria to 0.1 μM heme shortened the extended lag period that is otherwise observed when naive cells are inoculated into heme-containing medium, implying that GAS is able to adapt. The global response to heme exposure was determined using microarray analysis revealing a significant transcriptome shift that included 79 up regulated and 84 down regulated genes. Among other changes, the induction of stress-related chaperones and proteases, including *groEL/ES* (8x), the stress regulators *spxA2* (5x) and *ctsR* (3x), as well as redox active enzymes were prominent. The heme stimulon also encompassed a number of regulatory proteins and two-component systems that are important for virulence. A three-gene cluster that is homologous to the *pefRCD* system of the Group B Streptococcus was also induced by heme. PefR, a MarR-like regulator, specifically binds heme with stoichiometry of 1:2 and protoporphyrin IX (PPIX) with stoichiometry of 1:1, implicating it is one of the GAS mediators to heme response. In summary, here we provide evidence that heme induces a broad stress response in GAS, and that its success as a pathogen relies on mechanisms for heme sensing, detoxification, and repair.

## Introduction

Heme is vital to many biological systems, ranging from planktonic marine microorganisms to the highly complex and evolved humans. The heterocyclic ring in heme, protoporphyrin IX (PPIX), co-ordinates metal iron (Fe^+2^); this architectural arrangement supports a wide array of roles upon presentation of an appropriate microenvironment. Hemoglobin and myoglobin, for example, use heme as a prosthetic moiety for the transport and storage of oxygen and other diatomic gases. A multitude of redox enzymes engage heme as a catalyst of electron transfer. Heme is also associated with energy conservation and biotransformation in processes such as photosynthesis and respiration. In addition, signal transduction pathways are integrated via heme due to its ability to bind gaseous ligands such as oxygen, carbon dioxide, carbon monoxide, and nitric oxide (Heinemann et al., 2008; Chiabrando et al., 2014; Hogle et al., 2014). Notably, heme in hemoproteins constitutes a major iron reserve used by invading microorganisms during infection (Brown and Holden, 2002; Nobles and Maresso, 2011). Blood in particular is an immediate source of iron and heme for the majority of pathogens. Invading bacteria are often able to obtain heme directly from the circulating pool of hemoproteins. Some pathogens also deploy hemolysins to trigger erythrocyte destruction and hemoglobin release (Nobles and Maresso, 2011; Kozarov, 2012; Caza and Kronstad, 2013).

Ironically, while heme is essential for many functions, the transitional nature of the coordinated iron renders it a significant pro-oxidant that can harm many cellular entities including DNA, proteins, cytoskeleton, and membranes (Maines and Kappas, 1975; Solar et al., 1990; Chiabrando et al., 2014). To prevent cellular damage and to control infections, mammals restrict the pool of free circulating hemoproteins through scavenger proteins such as serum albumin, hemopexin (for heme), and heptoglobin (for hemoglobin) (Solar et al., 1989; Krishnamurthy et al., 2007). To minimize oxidative damage, the cellular levels of heme are managed by heme oxygenases. These enzymes degrade heme to biliverdin, which is then reduced to the cytoprotectant molecule bilirubin (Khan and Quigley, 2011). Since heme could act as a menace, it is advantageous only in amounts that meet the bacterial requirements and, as long as it remains sequestered, preventing access to susceptible macromolecules (Anzaldi and Skaar, 2010). Bacteria that are exposed to heme and take advantage of it during infection had to develop strategies to manage its toxic effects. While we do not fully understand how bacteria avoid and manage the negative ramifications of heme exposure, recent studies have begun to shed light on some of the molecular mechanisms that are involved in this process (Anzaldi and Skaar, 2010; Fernandez et al., 2010; Wakeman et al., 2014).

Heme tolerance in bacteria is typically based on tight regulation of heme uptake and degradation. Often bacteria employ Fur- or DtxR-like metallorepressors to orchestrate heme metabolism in response to iron availability (Nobles and Maresso, 2011; Wakeman and Skaar, 2012). In some cases, auxiliary defense systems that facilitate repair, detoxification, and expelling of heme surplus are activated when the cellular mechanisms for heme homeostasis are overwhelmed. A number of two-component systems (TCS) that coordinate the response to heme stress have been described in bacteria. The Heme Sensor System (HssRS) is a TCS that activates the expression of a heme efflux transporter (*hrtAB*) in *Staphylococcus aureus, Bacillus anthracis*, and possibly the Group B Streptococcus (GBS) (Torres et al., 2007; Stauff and Skaar, 2009; Fernandez et al., 2010). In response to heme pressure, the ChrAS TCS from *Corynebacterium diphtheria* inhibits the heme biosynthetic gene, *hemA*, and activates the transcription of the heme oxygenase gene, *hmuO*, required for heme catabolism (Bibb et al., 2005, 2007; Ito et al., 2009; Heyer et al., 2012). Interestingly, in addition to putative HssRS TCS, GBS also codes for *pefR*, a heme-responding repressor (Fernandez et al., 2010). PefR binding to heme leads to derepression of two heme export systems, namely *pefAB* and *pefCD* (Fernandez et al., 2010). It is suggested that GBS uses PefR to fine-tune heme homeostasis, while it deploys the HssRS system in response to high heme exposure.

As mentioned above, bacteria often detoxify, using active export systems that directly eliminate accumulated heme or its toxic byproducts. The efflux system, MtrCDE, in *Neisseria gonorrhea*, is crucial for bacterial resistance to hydrophobic agents and heme; overexpression of this machinery leads to increased bacterial burden in vaginal fluids (Hagman et al., 1995; Bozja et al., 2004). GBS mutants in *pefAB* or *pefCD* genes are over-sensitive to heme and demonstrate increased accumulation of intracellular heme and PPIX (Fernandez et al., 2010). Respiration-linked studies in *Lactococcus lactis* incriminated the *hrtAB* homologous genes *ygfAB* in heme efflux (Pedersen et al., 2008). A family of cytoplasmic proteins that bind and sequester heme forms another facet of heme tolerance. The proteins HemS of *Yersinia enterocolitica*, ShuS of *Shigella dysenteriae*, PhuS of *Pseudomonas aeruginosa*, and HmuS of *Y. pestis* are established members of this family (Stojiljkovic and Hantke, 1994; Thompson et al., 1999; Wilks, 2001; Wyckoff et al., 2005; Lansky et al., 2006; Lechardeur et al., 2010). Interestingly, the enzymes SodC of *Haemophilus ducreyi* and AhpC of the GBS have both heme sequestration roles in addition to enzymatic activity (Negari et al., 2008; Lechardeur et al., 2010). Bacteria also degrade heme to alleviate toxicity. Heme oxygenases were described in several pathogenic bacteria (Zhang et al., 2011; Uchida et al., 2012; Nambu et al., 2013; Wilks and Heinzl, 2014). Despite the clear role heme metabolism has in pathogenesis, our understanding of heme tolerance is lacking in numerous pathogens.

The Group A Streptococcus (GAS) is a Gram-positive obligate human pathogen. GAS infections range from mild diseases such as pharyngitis and impetigo to invasive and systemic manifestations, including necrotizing fasciitis and streptococcal toxic shock syndrome. GAS can also produce post-infection complications such as glomerulonephritis and rheumatic fever (Cunningham, 2008). In the absence of a vaccine, GAS infections are commonly treated with β-lactam antibiotics. However, due to the swift progression rate and extent of tissue damage, surgical intervention is often required to manage invasive GAS infections (Cole et al., 2011). The grave nature of invasive GAS infection is attributed to many virulence factors, including its β –hemolytic property that causes massive erythrocyte and tissue lysis (Nizet, 2002). GAS requires iron for growth and the pathogen can fulfill its needs for the metal by acquiring heme from lysing erythrocytes (Eichenbaum et al., 1996; Montanez et al., 2005). Heme acquisition is mediated by the *sia* (*streptococcal iron acquisition*) operon that facilitates heme relay and transport across the bacterial membrane (Bates et al., 2003; Liu and Lei, 2005; Nygaard et al., 2006; Sook et al., 2008; Ouattara et al., 2010). *In vivo* studies on a second operon named *siu* (*streptococcal iron uptake*) linked it to the uptake of heme and ferric ion (Montanez et al., 2005). Although heme receptors and transport proteins have been identified, our understanding of heme metabolism in GAS is still incomplete. The focus of this study was to fill in the knowledge gaps in GAS heme tolerance. Here, we show that excess heme is inhibitory for the growth of GAS *in vitro*; heme exposure caused a global transcriptome shift wherein it significantly up regulated genes that are important for redox stress, which includes sensing and management along with protein damage and rescue.

## Materials and methods

### Bacterial strains and growth conditions

Strains and plasmids used in this study are listed in Table 1. GAS was grown under static conditions at 37°C in Todd–Hewitt broth with 0.2% (wt/vol) yeast extract (THY broth, Difco Laboratories) or in C-medium consisting of 0.5% Proteose Peptone #3 (BD), 1.5% yeast extract (BD), 10 mM K_2_HPO_4_, 0.4 mM MgSO_4_, 17 mM NaCl, adjusted to pH 7.5 (Lyon et al., 1998). In some experiments, hemin chloride (Sigma) from stock solutions prepared in DMSO was added to the growth media at different concentrations. *E. coli* cells were used for cloning and protein expression purposes. *E. coli* was grown aerobically in a Luria–Bertani medium (pH 7.0) supplemented with kanamycin (30 μg/ml) at 37°C.

**Table 1:** **Strains used in this study**.

**Name**	**Description**	**References**
**STRAINS**		
MGAS5005	GAS (M1), isolated from cerebrospinal fluid	Sumby et al., 2005
NZ131	GAS (M49) isolated from acute post-glomerulonephritis infection	Mcshan et al., 2008
GA01398	GAS (M11).	GA-EIP^*^
GA06439	GAS (M114)	GA-EIP^*^
GA02581	GAS (M1)	GA-EIP^*^
GA02582	GAS (M1)	GA-EIP^*^
GA10156	GAS (M75)	GA-EIP^*^
COL (MRSA)	*S. aureus*, clinical specimen isolated from operating theater in England	Gill et al., 2005
*E. cloni*^®^ 10G	Host for pAJS11 propagation and expression	Lucigen
**PLASMIDS**		
pRham™ N-His	Protein expression vector, Kan^R^, expressed from rhaP_BAD_	Lucigen
pAJS11	Expresses N-Terminal His-tag PefR, KanR	This study

* *Georgia Emerging Infections Program (GA-EIP)*.

### Determination of the minimal inhibitory concentration (MIC)

#### Agar dilution method

This procedure was performed as described in Wiegand et al. (2008) with minor modifications. Briefly, the turbidity of overnight grown cultures in C-media was adjusted to OD_600 nm_ = 0.08–0.1, representing the turbidity of 0.5 MacFarland's acidified barium chloride standard as described (McFarland, 1907) and spotted (1 μL) onto C-media agar containing varying concentrations of heme. The plates were incubated overnight at 37°C and the minimal heme concentration that did not allow for significant bacterial growth was determined.

#### Broth macrodilution method

This method was performed as per the instruction of Wiegand et al. (2008). In brief, THYB containing heme at a range of 0–100 μM in 5 μM intervals was inoculated with GAS cells (OD_600 nm_ = 0.05) and incubated at 37°C for 17 h. The minimal heme concentration that did not support growth (OD_600 nm_ ≤ 0.2) was determined.

#### Disc diffusion method

This procedure was adapted from Drew et al. (1972). Briefly, sterile Whatman filter paper discs (8.0 mm diameter and 1.2 mm width) were submerged in 10 mM heme (in DMSO) and impregnated onto C-media agar that was plated with 0.1 ml of GAS culture (final OD_600 nm_ = 0.1). The plates were incubated at 37°C for 17 h and the zone of clearance around the discs were measured.

### Thiobarbituric acid-reactive-substances (TBARS) assay for lipid damage

GAS culture samples with equal cell numbers were harvested 30, 60, and 90 min following treatment with 4 μM heme (in 0.035% DMSO) or with mock (0.035% DMSO, control) and washed twice with phosphate buffer saline, pH 7.4 (PBS). The resulting cell pellets were resuspended in 5 ml of PBS with lysozyme (1 mg/ml, Sigma) and 400 U of mutanolysin (sigma) and incubated at 37°C for 30 min. The cells were then subjected to sonication (5 s, 10% amplitude). TBARS formation in the membrane samples was quantified using the TBARS assay kit (ZeptoMetrix Corporation) according to the manufacture's recommendations. Briefly, 100 μl cell lysate samples were treated with 100 μl of SDS and 2.5 ml of the TBA reagent and incubated at 95°C for 60 min. The sample supernatant was collected following 10 min incubation on ice by centrifugation (15 min at 835 × g). The absorbance at 532 nm (indicative of TBARS) was measured using the DU 730 Life Science UV/Vis spectrophotometer. The amount of TBARS in the experimental samples was derived from a standard curve generated using the malondialdehyde (MDA) reagent supplied with the kit.

### Detection of protein damage

The TBARS assay was followed as above except the cell pellets were washed twice with TSM buffer (100 mM Tris, 500 mM sucrose, 10 mM MgCl_2_, pH 7.0), resuspended in 0.5 ml of TSM with 400 U of Mutanolysin (Sigma) and incubated at 37°C for 30 min. The protoplasts were centrifuged at 20,000 × g for 5 min at 4°C, suspended in 0.2 ml of lysis buffer (50 mM Tris-HCl, 60 mM KCl, 10 mM MgCl_2_, pH 7.0, 2% β-mercaptoethanol) and subjected to sonication (10 s, 10% amplitude). The membrane components were collected by centrifugation at 100,000 × g for 30 min at 4°C. The supernatant was retained as a cytoplasmic fraction and pellets were resuspended in 0.2 ml of lysis buffer and were considered as the membrane fractions. Protein damage was detected using the OxyBlot™ protein oxidation detection kit (Millipore) according to the manufacturer's instructions. In this assay, oxidized proteins are derivatized with 2,4-dinitrophenylhydrazine (DNP), which is then detected with primary antibodies specific for DNP and secondary antibody conjugated to horseradish peroxidase (HRP) using a chemiluminescence protocol. The intensity of the signal in individual lanes was quantified using ImageQuant LAS 4000 and the ImageQuant TL software (GE).

### RNA extraction

Cell samples were harvested 30, 60, and 90 min following heme or mock treatment by centrifugation (5000 × g for 20 min) at 4°C. RNA was extracted from the cell pellet using the RiboPure™ RNA purification kit (Ambion) followed by DNaseI digestion (Ambion) performed according to manufacturer's instructions. Microarray analysis was performed with total RNA extracted from 90 min post treatment cell samples. Real-time RT-PCR transcript analysis for selected genes was performed using total RNA extracted from all three-time points post treatments.

### Microarray analysis and real-time RT-PCR validation

The GAS microarray used in this study (Ribardo and McIver, 2006) consists of 2328 70-mer oligonucleotide probes targeting unique non-repetitive ORFs from the sequenced genomes of serotypes M1 (SF370), M3 (MGAS315), and M18 (MGAS8232). Probes were synthesized by Qiagen Operon with a melting temperature of 76 ± 5°C, ≤70% cross-hybridization identity to another gene within the same strain, ≤20 contiguous bases in common with another gene, and probe location within 3′ end of ORF. Microarrays were printed at Microarrays Inc. (http://www.microarrays.com), with 10 pl of each oligonucleotide probe spotted onto slides (UltraGAPS2; Corning) using a 12-pin contact printer. The microarray hybridization was performed as previously described (Jiang et al., 2008). Briefly, 10 μg of DNase I-treated total RNAs to be compared were used for reverse transcription into single-stranded cDNA using 200 U Superscript II reverse transcriptase (Life Technologies), 6 μg random hexamers, 1X first strand buffer, 10 mM dithiothreitol (DTT), 0.5 mM dATP, 0.5 mM dCTP, 0.5 mM dGTP, 0.3 mM dTTP, and 0.2 mM of amino-allyl dUTP. The mixture was incubated at 42°C for 2 h and the reaction stopped by addition of 10 μl 0.5 M EDTA and 1 M NaOH. Amine-modified cDNA was purified by ethanol precipitation followed by chemical labeling with Cy3- or Cy5-NHS-ester fluorescent dyes (GE Healthcare) in a final step. Yield and incorporation of dye was determined using a Nanodrop ND-1000 (Thermo Scientific). Slides were pre-hybridized in a 50 ml solution of 5X SSC, 0.1% SDS and 1% BSA for 30 min at 42°C, washed 4X in water and once in isopropanol, then dried by brief centrifugation. Labeled probes were resuspended in hybridization buffer (30% formamide, 5X SSC, 0.1% SDS, 0.6 μg/μL salmon sperm DNA) and hybridized to the microarray slides in a 42°C water bath for 16–20 h. Slides were washed twice in a low stringency buffer (2X SSC, 0.1% SDS) at 55°C for 5 min, twice in a medium stringency buffer (0.1X SSC, 0.1% SDS) at room temperature for 5 min and finally twice in a high stringency buffer (0.1X SSC) at room temperature for 5 min, and then dried by brief centrifugation. Synthesized cDNA from each RNA sample from three independent cell cultures was hybridized on three separate microarray slides (biological replicates), and independently synthesized cDNA from each of these same RNA samples was hybridized in a repeat dye-swap experiment (technical replicates) to test technical reproducibility. Slides were scanned using an Axon 4100A personal array scanner and GenePixPro 6.0 software (Molecular Devices). Data obtained from MGAS5005 cells incubated in the presence of DMSO or 4 μM of heme were compared for twofold changes in expression, ≥2.0 or ≤0.50 with Acuity 4.0 software (Molecular Devices). Using a ratio-based normalization, data were normalized by the ratio of the means (635/532) and samples were removed when four out of the six experiments did not show significance. Data was validated on 11 independent genes by real-time RT-PCR as described below. Correlation coefficients for the arrays were determined by plotting the log value of the array on the x-axis to the log value of the real-time RT-PCR on the y-axis. An equation determining the line of best fit was determined, and the resulting R^2^ value was calculated to be 0.889. Array data was submitted to the Gene Expression Omnibus (GEO) at the National Center for Biotechnology Information under the accession number GSE61415.

### Quantification of expression by real-time RT-PCR

For microarray data validation, real-time RT-PCR analysis was carried out using primers in Table 2 as follows: 25 ng of DNaseI-treated total RNAs were added to SYBR green master mix (AB) containing 200 nM of each specific real-time primer for the one-step protocol. The real-time RT-PCR experiments were completed using a LightCycler 480 instrument (Roche), and levels represent the ratio of non-treated to treated experimental values relative to the level of expression of *gyrA* transcript as an endogenous control. For expression of the *pefRC* operon, a SYBR Green based quantitative PCR reactions were performed using the Power SYBR® Green RNA-to-Ct™ 1-Step Kit (AB) on 7500 Fast Real-Time PCR machine (AB) according to the manufacturer's specifications. Briefly, the reaction mixture (20 μl) contained, 10 μl of RT-PCR Mix (final 1X), 200 nM of each forward and reverse primers, 25 ng of total RNA, and RT enzyme Mix in 1X final concentrations. The relative quantification with comparative ΔΔCT method was employed to calculate differences in *pefRC* expression levels at different times after heme exposure. The relative expression levels of *pefR* and *pefC* genes were normalized to the level of *rpsL* transcript as an endogenous control. The levels of transcripts in heme treated samples were compared to the control samples.

**Table 2:** **Primers used in this study**.

**Target**	**PCR primers**	**Sequence (5′-3′)**
*pefR* ORF	ZE515	CATCATCACCACCATCACTCACAAGTGATAGGTGATTTACG
	ZE516	GTGGCGGCCGCTCTATTAAGCATCGTTGTCTCCTTTATAA
P_*pef*_	ZE561	AAGGCGTTCCCAAGAGAGCTAG
	ZE562	CCTTGAGGACCTGCTAGATGCTCTAC
*pefR*	ZE569	CAATGTGATGCCTTCCCAAG
	ZE570	CGCTGTCAGCAACTCTTC
*pefC*	ZE571	CCTCATCATGGGTTTGGTG
	ZE572	ACGCCACGGAGATTTTCC
*rpsL*	ZE581	CAGATTCACCAGCTTTGAAC
	ZE582	CAACACGAGTAGCAACG
prsA	prsA-M1-RT-L	GGGCAGACTTTGCAGCTATTG
	prsA-M1-RT-R	TCGCCTGAGTCAAACGTATAGG
*clpL*	clpL-M1-RT-L	TGGCTTGAGCTAAACCTTCA
	clpL-M1-RT-R	CTTGGCACGACGAACTAAAA
*opuAA*	opuAA-M1-RT-L	TGATTTGCAAGACAGCATGA
	opuAA-M1-RT-L	CATCAAAGCAATCCGATCAC
*endoS*	endoS-M1-RT-L	CTCGGTCAATAGCGTAGGAGAAG
	endoS-M1-RT-L	GCGTGCCGAACGGTATG
*ptsA*	ptsA-M1-RT-L	TTTTTTAAAACCAGGCGAAGC
	ptsA-M1-RT-L	TTGTCTCAGGGACCAAATCC
*sagA*	sagA-M1-RT-L	GCTACTAGTGTAGCTGAAACAACTCAA
	sagA-M1-RT-L	AGCAACAAGTAGTACAGCAGCAA
*spt7R*	spt7R-M1-RT-L	TCATTTGCGGCTGAAATAATAATG
	spt7R-M1-RT-L	GCAATGGGATTCAATTTTTGGA
*slo*	slo-M1-RT-L	TTGTTGAGGATAATGTAAGAATGTTTAG
	slo-M1-RT-R	TCCTGGCTTGCAACTGATTG
*fasC*	fasC-M1-RT-L	TGCGCACAAATTATGAAATATCTTC
	fasC-M1-RT-R	GAGCTTCAAGCAATTTGGAATTC
*mtsA*	mtsA-M1-RT-L	TGAGGGTCTTGACCGATTG
	mtsA-M1-RT-R	AAGTCGTGGCAACCAATTC

### *In silico* analysis

The PefR binding motif was identified within the putative promoter region of MGAS5005 *spy_0195* (*pefR*) using Multiple Em for Motif Elicitation (MEME) suit hosted by the National Biomedical Computation Resource (Bailey et al., 2006). The outcome was further analyzed by the MAST application of MEME for its genome wide occurrence within the upstream sequences of GAS under both the stringent (*E*-value ≤ 0.01) and relaxed (*E*-value ≤ 10) parameters. All of the sequences were acquired from Kyoto Encyclopedia of Genes and Genomes (KEGG) database (Kanehisa, 2002) for comparative sequence analysis; sequences were aligned using ClustalW (Thompson et al., 2002).

### Cloning, overexpression, and purification of PefR

The *spy_0195* ORF was amplified from GAS MGAS5005 genomic DNA by PCR using primers ZE515 and ZE516 (Table 2). To generate pAJS11 plasmid (Table 1), the PCR fragment with *pefR* ORF was cloned into the pRham™ expression vector (Lucigen) by Expressioneering™ technology and introduced into *E. cloni®* 10G competent cells. The cloning was confirmed by sequence analysis. For PefR expression, cells harboring pAJS11 were induced at OD_600 nm_ = 0.8 with 0.2% rhamnose. The cells were harvested (8000 × g for 5 min at 4°C) following 16 h incubation at 28°C. The resulting pellets were resuspended in extraction buffer (20 mM Tris pH 8, 100 mM NaCl, 0.1% Triton X-100) containing 0.5 mg/mL Complete, mini-EDTA-free protease inhibitor cocktail (Roche), sonicated and the cell debris were removed by centrifugation. The resulting lysate was purified over 5 ml HisTrap™ HP (GE) nickel affinity column. Protein fractions were dialyzed overnight in sodium phosphate buffer (SPB: 20 mM sodium phosphate, 500 mM NaCl pH 7.4). Expression of the recombinant PefR was evaluated by SDS-PAGE and western blot analysis with mouse anti-His antibodies (Sigma).

### Heme and PPIX binding assay

Heme binding by PefR was assessed spectroscopically as described in Puri and O'brian (2006) and Ouattara et al. (2010) with minor modifications. Briefly, an increasing concentration of hemin chloride (2–30 μM) was added to both the test cuvette containing 10 μM of PefR protein in SPB and the reference cuvette (containing SPB alone) and the changes in absorbance across the wavelength of 250–700 nm region were recorded every 6 min. The PefR to heme stoichiometry and dissociation constant (*K_d_*) were determined by plotting the absorbance at 435 nm as a function of the heme concentrations. The extinction coefficient (ε_max_) for PefR was calculated from the hemocromogen method described in Asakura et al. (1964). All of the spectroscopic measurements were made using the DU730 Life Science UV/Vis spectrophotometer (Beckman Coulter). PPIX binding was tested by titrating PefR (5 μM) with 0–6 μM PPIX (in 0.5 μM increments) prepared in acidified methanol:dimethylformamide (1:1). The changes in absorbance were recorded.

## Results

### Excess of heme is inhibitory to the growth of GAS

Host heme is the immediate source of iron during infection for invading pathogens such as GAS. Recent studies demonstrated that bacteria must maintain the intracellular levels of heme at equilibrium in order to benefit from its nutritional value, while eluding the toxicity that is associated with heme overload (Torres et al., 2007; Fernandez et al., 2010; Mike et al., 2014). In this work, we began evaluating the impact of heme on GAS physiology. We found that the addition of a disc saturated with 10 mM heme onto agar plates seeded with a confluent lawn of GAS resulted in a zone of clearance similar to those observed with antibiotic-impregnate discs. This observation indicates that heme can have a bacteriostatic effect on GAS (Table 3). To compare the sensitivity to heme among GAS isolates we determined the MIC values of heme using two common methods (Table 3). The agar dilution assay employs solid C-medium containing varying concentrations of heme. We determined that the heme MIC in this method is between 10 and 20 μM with the nephritogenic M49 GAS skin isolate, NZ131 (Simon and Ferretti, 1991; Mcshan et al., 2008), exhibiting the highest sensitivity of all GAS strains tested. The highly pathogenic M1T1 MGAS5005 strain (Sumby et al., 2005) as well as clinical isolates from patients with invasive diseases (Table 1) demonstrated higher heme tolerance. In the broth dilution assay, we measured bacterial growth in THYB supplemented with varying heme concentrations. This method resulted in higher values of heme MIC (30–50 μM) for MGAS5005 and the clinical isolates. However, both methods indicated that the invasive strains were more resistant to heme than NZ131.

**Table 3:** **Bacteriostatic effect of heme**.

**Strain^a^**	**Agar dilution (μM)**	**Broth dilution (μM)**	**Disc diffusion (mm)**
NZ131 (M49)	10	10	9
GA01398 (M11)	20	30	1.25
GA06439 (M114)	20	30	6.75
GA02581 (M1)	20	50	6
GA02582 (M1)	15	50	4.75
GA10156 (M75)	20	30	4.5
MGAS5005 (M1)	20	50	5.5
*S. aureus (control)*	260	80	4.25

*The minimal heme concentration that inhibits bacterial growth (MIC) was determined by the methods of agar dilution and broth macrodilution. The growth inhibitory effect of heme is also expressed as the clearance zone around heme impregnate discs (10 mM). See material and methods for details. Data are representative of two independent replicates*.

a *GAS M type (if available) is indicated in parentheses*.

### Exposure to sub-lethal heme levels cause lipid peroxidation in GAS

**Figure 1:**
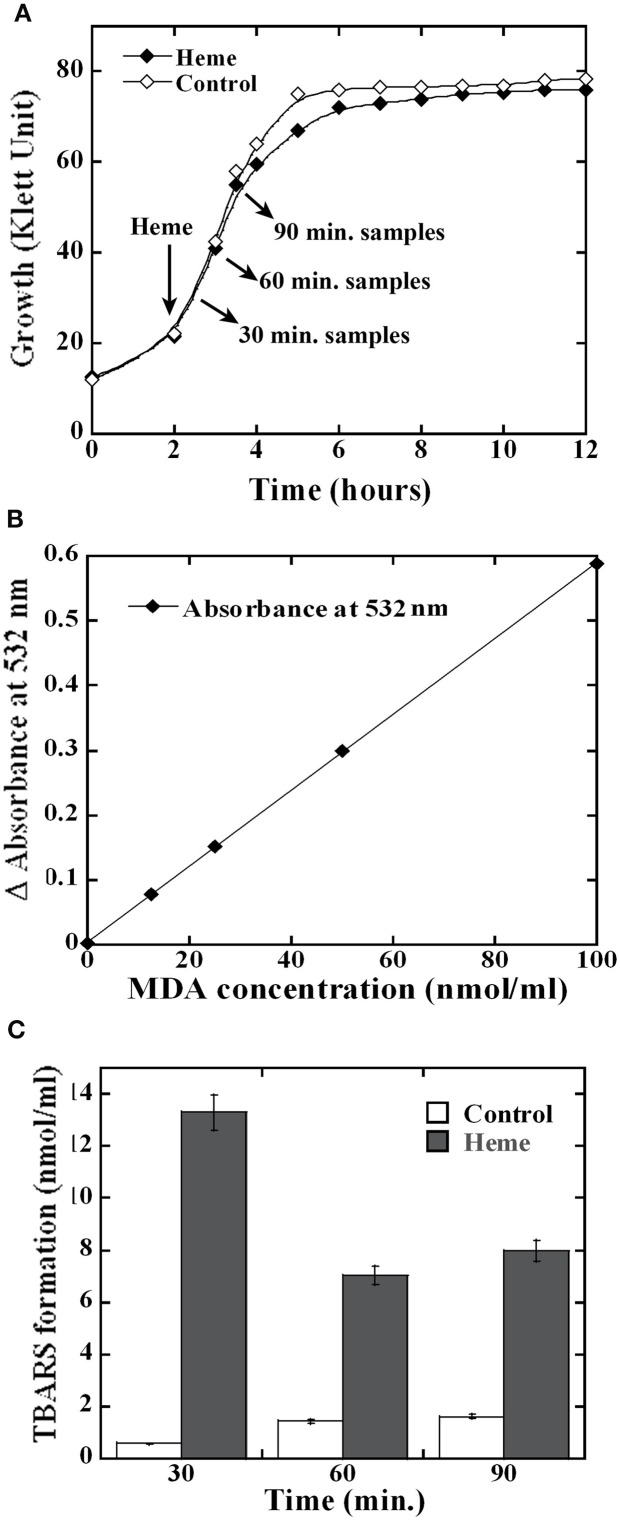
**Heme exposure leads to lipid peroxidation in GAS**. **(A)** Cell growth. MGAS5005 cells growing in C-media were treated with 4 μM heme in 0.035% DMSO (Heme) or with mock treatment (0.035% DMSO, Control) at the early logarithmic growth phase. Culture samples were harvested 30, 60 and 90 min post exposure and processed. **(B)** Malondialdehyde (MDA) standard curve. 100 μl samples of MDA at a concentration of 0, 12.5, 25, 50, and 100 nmol/ml were allowed to react with thiobarbituric acid (TBA, see Materials and Methods). The absorption at 532 nm of the supernatants from processed samples was determined and plotted as a function of MDA concentration exposure. The linear equation obtained for the standard curve was *y* = 0.0059 × with R^2^ value of 0.9997. **(C)** Lipid peroxidation following heme exposure. Cell lysates were prepared and allowed to react with TBA. The sample absorption at 532 nm was determined and the formation of TBA-reactive-substances (TBARS) was calculated using the standard curve shown in **(B)**. All samples were standardized with respect to the cell number in the corresponding culture. Data are representative of biological triplicates.

To learn why heme is detrimental for GAS growth, we investigated its impact on GAS macromolecules. Heme is reported to damage directly or through the generation of reactive oxidative species the integrity of membrane lipids (Chang et al., 2003; Chiabrando et al., 2014). Therefore, we evaluated the extent of lipid peroxidation in GAS following heme exposure. Using low heme concentration that could still support cell growth when applied at the early logarithmic phase allowed us to examine the kinetics of damage production and later the bacterial transcriptome response to heme. As seen in Figure 1A, the addition of 4 μM heme to MGAS5005 at the early logarithmic phase of growth did not result in a significant change in the bacterial growth profile. To detect lipid peroxidation, we used an established method that relies on the tendency of oxidized lipids to react with a thiobarbituric acid (TBA) reagent to form adducts (named TBARS) that absorb at 532 nm (Hong et al., 2012). The time course of TBARS production in GAS cells exposed to heme (or in control samples) was quantified using a standard curve (Figure 1B). Significantly higher occurrence of lipid peroxidation was observed in all of the heme-treated samples compared with control samples (Figure 1C). Lipid peroxidation was the highest (22.5 fold over background) 30 min after heme treatment; while lower levels (~5 fold over background) were found in the samples collected 60 and 90 min post-heme treatment (Figure 1C), indicating possible repair mechanism. In eukaryotes, membrane repair involves enzymes such as phospholipase A2 that hydrolyse peroxidized fatty acid esters and glutathione peroxidase (van Kuijk et al., 1987; Thomas et al., 1990).

### Exposure to heme damaged membrane proteins

**Figure 2:**
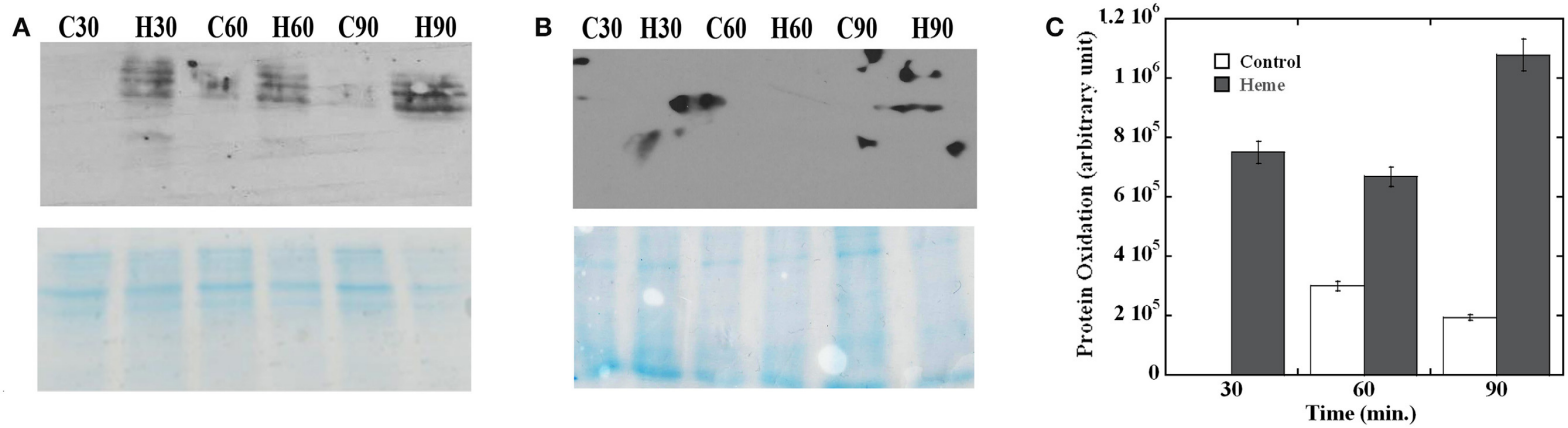
**Protein oxidation following heme exposure**. The membrane **(A)** and cytoplasmic proteins **(B)** extracted from cells harvested at different time points post treatment were allowed to react with DNPH (see Materials and Methods) before fractionation by SDS-PAGE. Sample identity (H for heme treated cells and C for controls) and collection times (30, 60, and 90 min) are indicated (top panel). Western blot with anti-DNP antibodies (OxyBlot) developed by chemiluminescence (bottom panel). Coomassie blue-stained gel. All samples were standardized with respect to the cell number in the corresponding culture. **(C)** Quantification of protein oxidation. The reaction with anti-DNP antibody in each membrane sample was quantified by densitometry. Arbitrary values for the heme treated samples (Heme) and mock treated (Control) after background subtraction are plotted. Error bars are shown. Data are representative of biological triplicates. Note that the western blot showing larger spots in an area outside of the lane is a technical artifact associated with the film development and it's not a true reaction (prominent in **B**, top panel).

We next evaluated the effect of heme on GAS proteins. Oxidation introduces carbonyl groups into protein side chains that can then react with 2,4-dinitrophenylhydrazine (DNPH) to generate 2,4-dinitrophenylhydrazone (DNP) derivatives (Dalle-Donne et al., 2003). To monitor protein oxidation in GAS, membrane and cytoplasmic proteins were extracted from culture samples collected at 30, 60, and 90 min post treatment and allowed to react with DNPH. Oxidation was then visualized by immunoblot with antibodies specific for DNP and the damage was quantified by densitometry. Analysis of the membrane fractions revealed that the anti-DNP antibody strongly reacted with all of the samples from heme exposed cells (Figure 2A, upper panel). While general staining confirmed the presence of proteins in all samples (Figure 2A, bottom panel), protein oxidation was only observed in the heme-treated cells in the early sample. At the later time points (60 and 90 min) some background oxidation also occurred in the control samples. In all cases though, a significantly higher amount of protein damage was observed in the heme-exposed cells compared to the controls (Figure 2C). In addition, the degree of protein oxidation was the highest in the 90 min samples. Similar analysis of cytoplasmic proteins did not reveal detectable damage (Figure 2B); therefore, protein oxidation following heme exposure is primarily linked to the membrane fraction under our experimental conditions.

### GAS demonstrates adaptation response to heme

**Figure 3:**
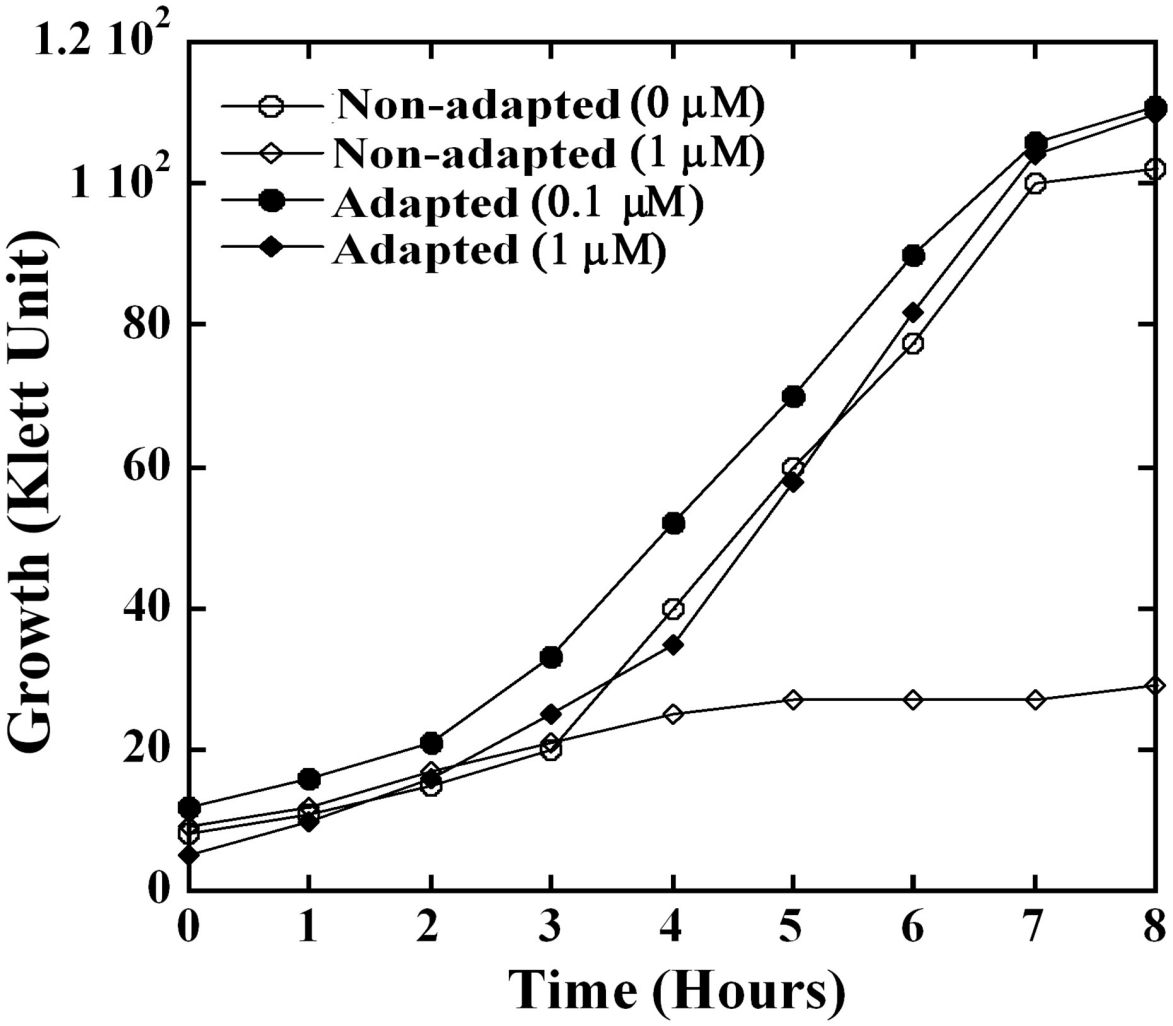
**GAS employs an adaptation strategy to avert growth phenotypes associated with heme stress**. NZ131 cells were grown overnight in C-media supplemented with 0.1 μM (adapted culture) and without heme (non-adapted culture). The overnight cultures were used as an inoculum to monitor growth in presence and absence of heme. Briefly, non-adapted culture was grown in C-media supplemented with 0 and 1 μM heme, whereas adapted culture was sub-cultured into C-media containing 0.1 and 1 μM heme. The growth was monitored colorimetrically for 8 h at 37°C.

When overnight cultures of NZ131 grown in heme-free medium were inoculated into medium containing 1 μM heme, which is below the growth inhibiting concentrations at the early logarithmic phase, an extended lag phase that lasted for more than 8 h was observed (Figure 3). Although exposure to heme inhibited growth initially, the cultures reached after overnight incubation the cell density observed in cultures that grew in heme-free medium (data not shown). To test for possible adaptation to environmental heme, GAS cells were grown overnight in the presence of 0.1 μM heme before they were inoculated into medium with 1 μM heme. Indeed, pre-exposure to low heme level eliminated the extended lag period (Figure 3). These observations suggest that the treatment with low level of heme-triggered adaptation to environmental heme.

**Figure 4:**
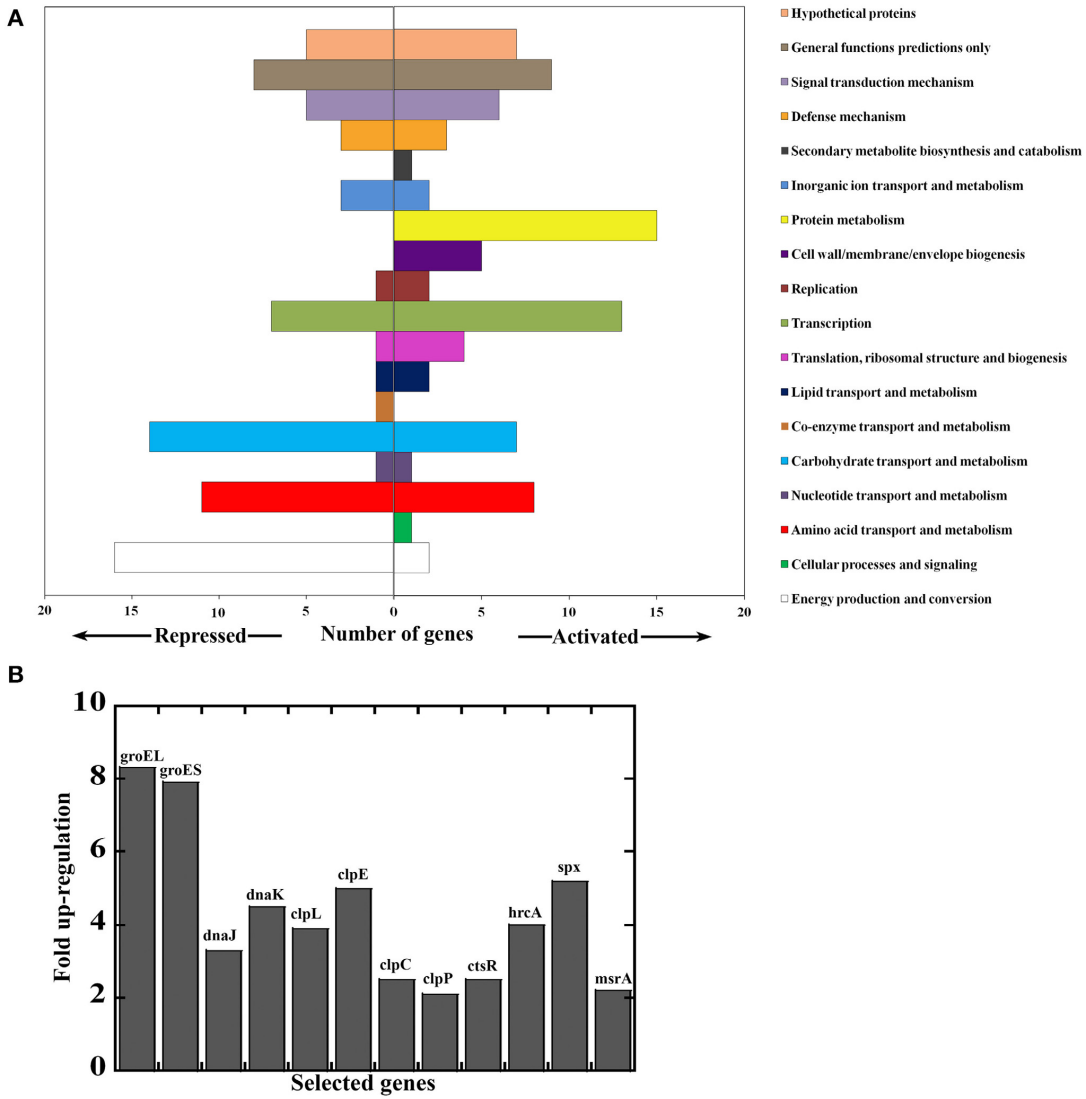
**Genes responding to heme stress in M1T1 GAS strain MGAS5005**. Total RNA was extracted 90 min post treatment and analyzed by microarray. **(A)** Genes repressed or activated following exposure to 4 μM heme (in 0.035% DMSO) compared with mock treatment (0.035% DMSO). Data for genes whose expression was significantly different between cells exposed to heme and those subjected to mock treatment were combined and assigned to categories. **(B)** Selected heme-activated genes revealing protein and redox stress response. Values represent relative expression levels (fold-change) in heme treated bacteria compare with mock treatment.

In light of recent evidence that bacteria can sense and respond to heme (Fernandez et al., 2010; Lechardeur et al., 2012; Mike et al., 2014; Wakeman et al., 2014) and with evidence for a similar response in GAS, we wanted to gain insight into the pathogen's mechanisms for heme sensing and tolerance. We examined the differential global gene expression pattern between GAS cells subjected to heme stress (4 μM) and control treatment. Total RNA was isolated from cells 90 min post treatment and analyzed using a 70-mer oligonucleotide microarray (representing GAS genomes M1, M3, and M18) (Ribardo and McIver, 2006). Our data showed a significant shift in the transcript profile of different genes and operons in response to heme stimulus; referred to as the “GAS heme stimulon.” This stimulon involved changes in 163 total genes, including 79 genes that were up regulated and 84 genes that were down regulated (Figure 4 and Table S1). The array analysis was validated by quantitative RT-PCR (qPCR) on 11 differentially regulated genes (Table S1), showing a strong correlation, with an R^2^ value of 0.889 (Figure S1).

Genes that were down regulated significantly in response to heme are found in the categories of metabolic pathways, sugar and metabolite transport, and two-component system (TCS, Figure 4A). Of particular interest is the down regulation of the TCS, *trxSR*. The response regulator, TrxR activates the Mga virulence regulon in GAS, and a *trxR* mutant is attenuated for virulence in a murine infection model (Leday et al., 2008; Baruch et al., 2014). Therefore, environmental heme may impact the expression of key virulence factors in GAS via the TrxSR/Mga pathway. Since the TrxSR system is implicated in asparagine sensing, our observations also imply that the damage to the cell envelope introduced by heme is associated with increased levels of asparagine (or other amino acids) (Baruch et al., 2014). In contrast, heme-activated genes and highly expressed transcripts encode for protein folding and degradation (such as *groEL/ES* ~8 fold, *DnaJ/K* 3-4 fold, and *clpE/L* ~4 fold) and components of the Clp protease machinery (*clpCP*- 2.5 fold) (Lund, 2001; Alexopoulos et al., 2012) (Figure 4B). In addition, the GAS heme-activated stimulon includes genes whose functions are linked to regulation of redox stress management, including *spxA2* (5 fold) and *ctsR* (2.5 fold) (Elsholz et al., 2010; Antelmann and Helmann, 2011). Accordingly, members of the downstream genes of these regulators were also activated by heme such as thioredoxin (*trx* ~2.5 fold) and oxidoreductases (3 fold). Finally, the expression of two TCSs was also up regulated in response to heme exposure, including the pneumococcal-like TCS homologous system, *ciaHR* (~2.3 fold) (Ahn et al., 2006) and the *ihk/irr* TCS (2.5 fold) involved in GAS response to oxidative stress and survival within phagocytes (Han et al., 2012).

### Identification of the *pefRCD* locus in GAS

**Figure 5:**
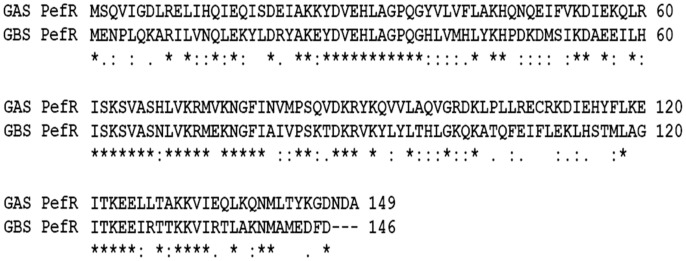
**Multiple sequence alignment of PefR amino acid sequence from GBS and GAS**. The degree of homology between GAS PefR (encoded by MGAS5005 *spy_0195*) and GBS PefR (encoded by *gbs1402*) is depicted in pair-wise sequence alignment performed using ClustalW. The amino acids are represented by single letter code. The symbols: (^*^) indicate identical residues; (:) indicate strongly similar residues; (.) designate weakly similar residues. An accuracy of alignment was also confirmed using MUSCLE tool (Edgar, 2004) that showed identical alignment output as well.

**Figure 6:**
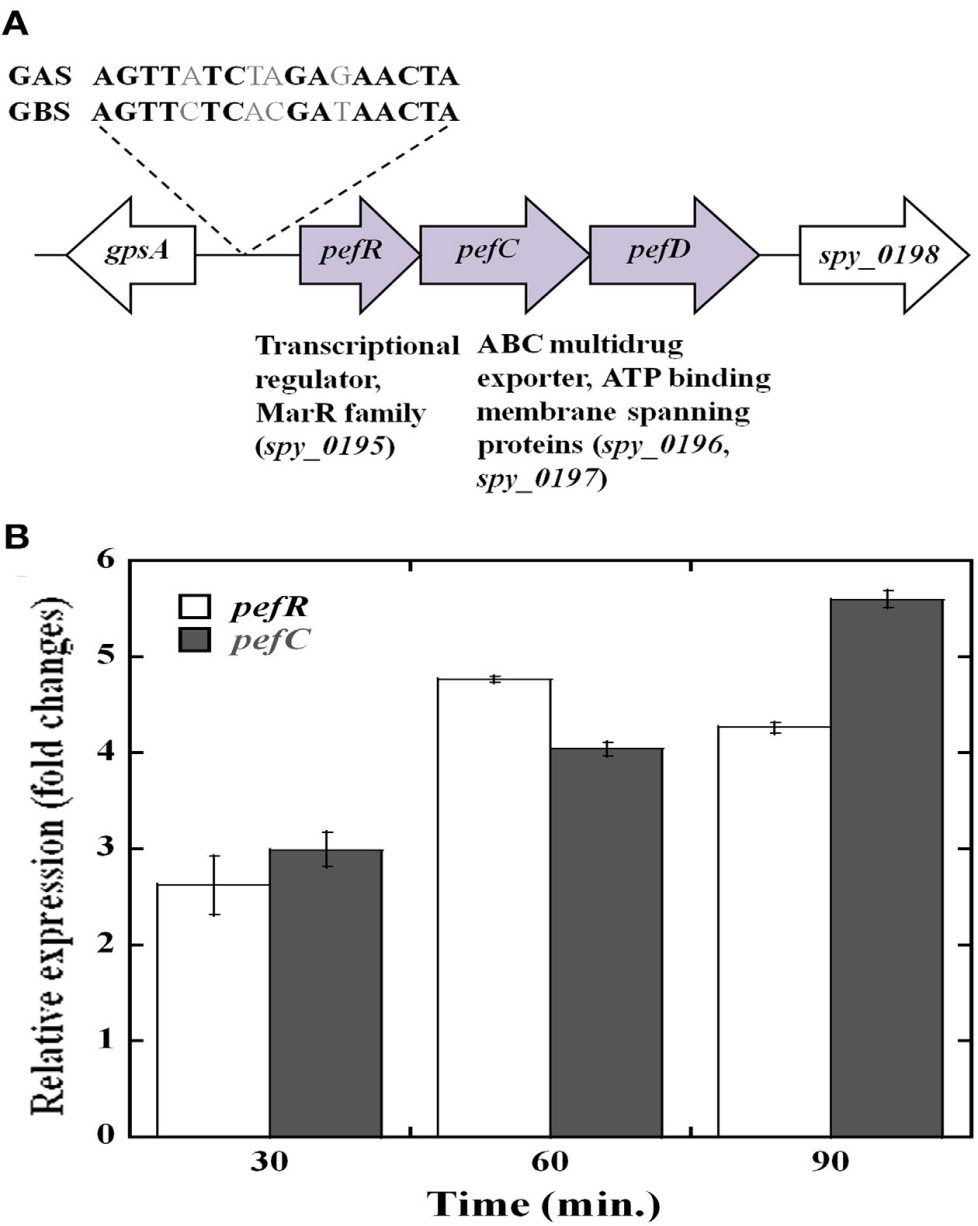
**The streptococcal *pefRCD* locus**. **(A)** Genetic organization of the *pefRCD* gene cluster in GAS. The putative PefR binding identified *in silico* and its homology to the PefR binding motif from GBS (Fernandez et al., 2010) are shown. **(B)** Time course of *pefRC* expression in response to heme stress. Total RNA was extracted at different time points following heme or mock treatment and the relative expression of the *pefR* and *pefC* genes was evaluated by Real-time quantitative RT-PCR.

The transcriptomic analysis also revealed that several putative efflux proteins also showed an increase in transcriptional activity in response to heme stress. These included a 3-gene cluster (MGAS5005 *spy_0195, 0196*, and *0197)* with homology to the *pefRCD* genes from GBS (Fernandez et al., 2010). Since the *pefRCD* genes are important for the management of heme stress in GBS, we focused our attention on their heme-induced homologs in GAS. Comparative sequence analysis demonstrated that the putative GAS genes, *spy_0195* (84% similarity; 56% identity), *spy_0196* (76% similarity; 48% identity), and *spy_0197* (76% similarity; 51.3% identity) show significant sequence homology to the GBS *pefRCD genes*, respectively (Figure 5). In addition to the high sequence homology demonstrated by each of these GAS genes, the genomic arrangement of the GBS *pefRCD* locus and its immediate chromosomal location are also conserved in all of the published GAS genomes (Figure 6A). Moreover, using the MEME algorithm, we identified a 17-bp inverted repeat region within the putative promoter of *spy_0195* that shared 76% identity with the PefR binding site from GBS (Figure 6A). Based on this sequence conservation and the observed induction by heme, we hypothesized that like GBS, the above-mentioned GAS genes encode a heme dependent repressor (*spy_0195*) and an ABC heme exporter (*spy_0196* and *spy_0197*) and named them *pefRCD*, respectively.

We examined the time course of *pefRC* expression in response to heme by qPCR (Figure 6B). As seen in the microarray analysis, the expression of *pefR* and *C* was comparable to one another in all of the time points, supporting an operon structure. Heme exposure resulted in an increase of *pefR* and *pefC* expression over time, reaching 2.5 fold over background at 30 min, 4.5 fold at 60 min and remained high at 90 min post treatment. This temporal increase in *pef* expression in response to heme insinuates an active participation of these genes in heme tolerance.

### GAS PefR binds heme and PPIX with high affinity

**Figure 7:**
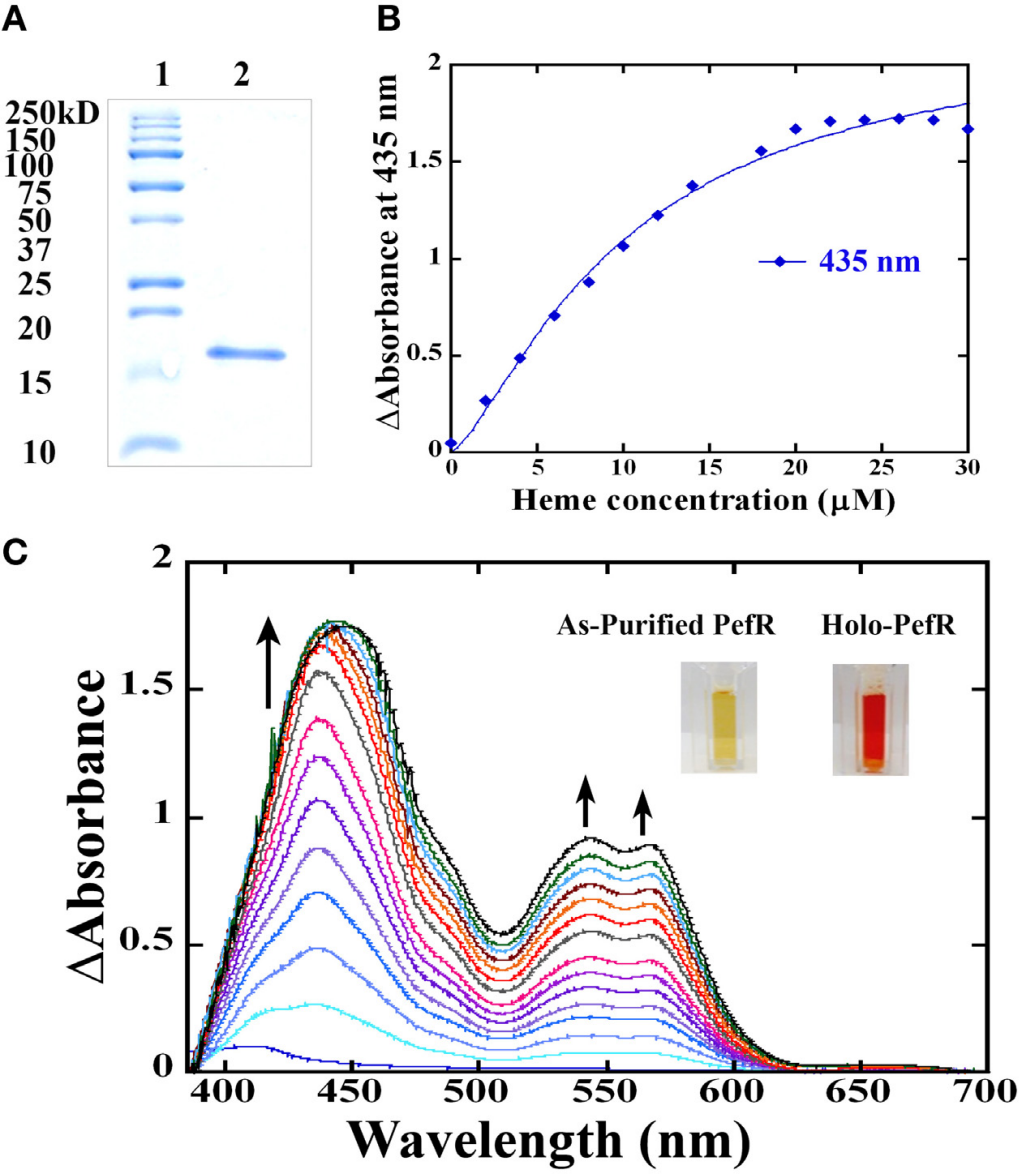
**The PefR from GAS is a heme binding protein. (A)** A Coomassie blue-stained gel showing purified recombinant PefR from GAS next to a molecular marker. **(B)** Heme titration of PefR. UV-visible spectrum of 10 μM PefR (in SPB) following incubation with an increasing concentration of hemin chloride (2–30 μM) in 2 μM increments. SBP with the corresponding heme concentration was used as blank and subtracted from the sample spectrum. Heme binding by PefR is indicated by the increased absorption at 435, 530, and 560 nm. The arrow indicates the direction of the absorption changes. The images are of PefR as-purified from *E. coli* and of holo-PefR (after heme reconstitution and removal of heme excess by gel filtration and dialysis). This data represents three independent heme-titration experiments. **(C)** Stoichiometry of heme binding to PefR. The changes in absorbance at 435 nm were plotted against heme concentration. The *Kd* was determined by fitting the data into modified hills equation for multiple binding sites (Goutelle et al., 2008). The *Kd* equals 10 μM and *n*H (hill's coefficient) is 1.37 μM.

**Figure 8:**
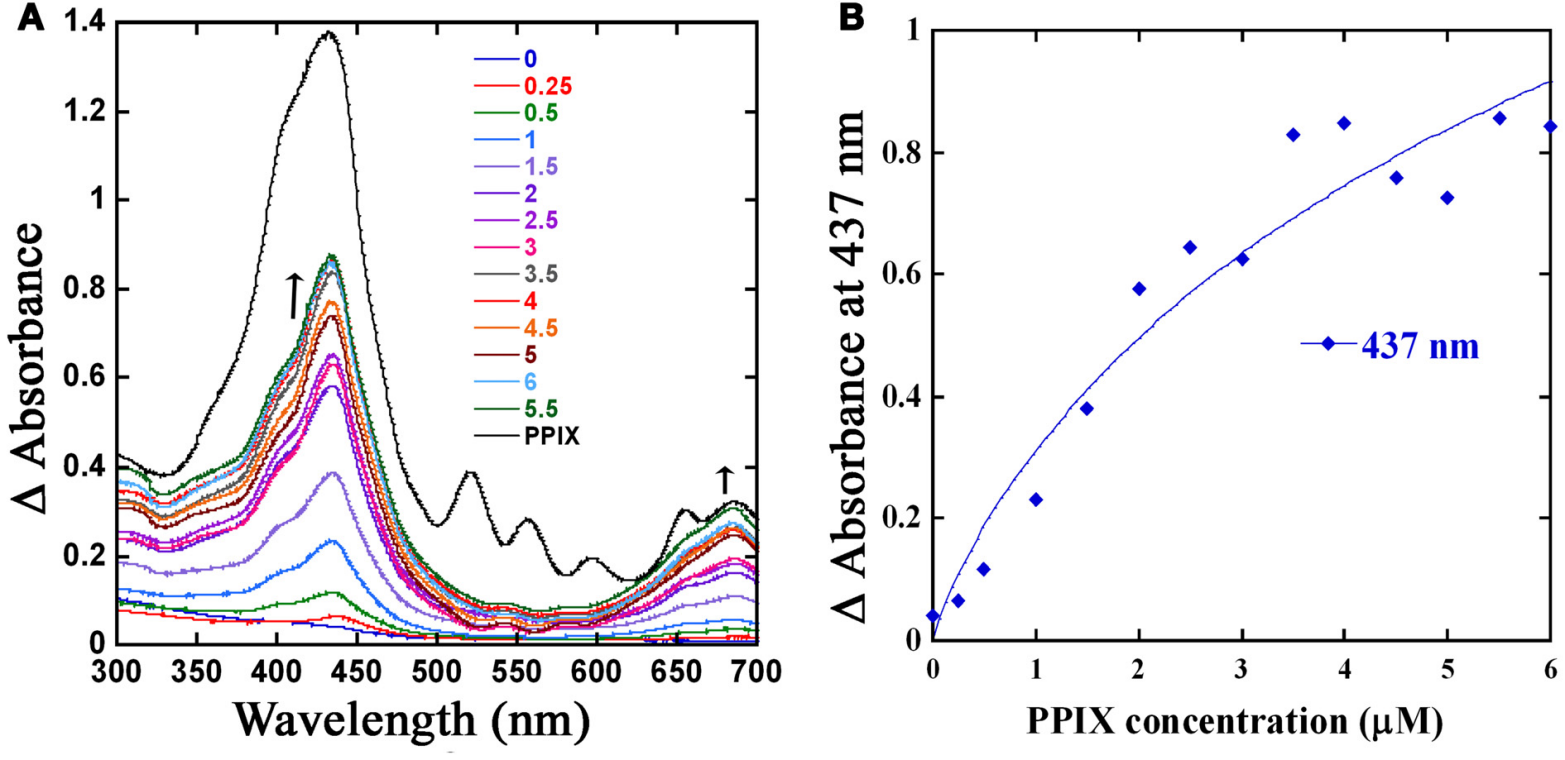
**The PefR is capable of PPIX binding. (A)** PPIX titration of PefR. Change in the UV-visible spectral profile of 5 μM of PefR (in SPB) when titrated with an increasing concentration of PPIX (0.25–6 μM) in 0.5 μM increments was monitored across wavelength (300–700 nm). Free PPIX spectrum (shown in black) showed absorbance peaks in 432, 521, 556, 596, 655, and 685 nm regions. The PefR bound PPIX demonstrated maximum absorbance at 437 and 686 nm; absorbance at these wavelengths showed increase with PPIX concentrations (0.25–6 μM; shown in different colors). The arrow indicates the direction of the absorption changes. **(B)** Stoichiometry of PPIX binding to PefR. The spectral changes at 437 nm were plotted against PPIX concentration. The *Kd* was determined by fitting the data into linear equation.

In GBS, PefR is a MarR-like repressor that binds to DNA and blocks the transcription of both *pefRCD* and *pefAB* operons. In this system, heme acts as an inducer that binds to PefR protein to alleviate repression. The GAS protein shares 43% identity and 74% similarity with PefR from GBS (Figure 5), implying the two proteins may function in a similar manner. We cloned the *pefR* gene from GAS and expressed it as an N-terminal hexahistidine (6x-His) fusion protein. SDS-PAGE and western blot analysis of the recombinant protein following purification from *E. coli* confirmed the presence of a single protein at the expected size that reacts with anti-his tag antibodies (Figure 7A and data not shown). Interestingly, the purified PefR had a light brown color (Figure 7B, left inset) and exhibited an absorption in the Soret region (435 nm). Upon titration with free heme, PefR displayed a growing Soret band and concomitant increase in absorption at 530 and 560 nm (β and α bands of heme). These spectral features are characteristics of heme bound protein and are easily separated from the absorption displayed by heme that is free in solution (Zhu et al., 2000). The holo-PefR maintained a bright red color after the removal of heme excess by gel filtration and dialysis (Figure 7B, right inset). Therefore, PefR was purified from *E. coli* with some heme and readily bound heme *in vitro*. The plot of changes in absorbance at 435 nm as a function of heme concentrations indicated a binding stoichiometry of 1:2 (PefR:heme, Figure 7C). We determined the dissociation constant (*Kd*) for PefR by fitting the plot of changes at 435 nm in the modified Hill's equation (Goutelle et al., 2008). We found the *Kd* to be 10 μM and Hill's coefficient (*nH)* to be 1.3, greater than 1 (Yifrach, 2004). The molar extinction co-efficient at 435 nm of holo-PefR was determined using the pyridine hemocromogen method (Asakura et al., 1964) and resulted in ε_435_ = 30,407 M^−1^cm^−1^. Similarly, titration of PefR with PPIX indicated that it could also bind PPIX in 1:1 stoichiometry with *Kd* of 9 μM (Figure 8).

## Discussion

It is the biochemical properties of heme that contribute to its characteristics as a double-edged sword in a variety of biological systems. Heme toxicity in humans stems from the lysis of erythrocytes due to disease, inflammation, or physical damage, which could raise the heme levels in the bloodstream up to 20 μM (Arruda et al., 2004; Kumar and Bandyopadhyay, 2005). The generation of reactive oxygen species, alteration in membrane permeability, damage to macromolecules, and decrease in the pool of reductants are some of the cellular mayhem that follows heme overload in eukaryotes (Kumar and Bandyopadhyay, 2005). In bacteria, despite the importance of heme to physiology and pathogenesis, the molecular mechanisms for heme toxicity and tolerance are poorly understood (Anzaldi and Skaar, 2010). In this study, we probed these processes in GAS for the first time and established that heme stress is very significant to the physiology of the β-hemolytic pathogen; heme in excess inhibits GAS growth and exposure to low heme levels damages membrane lipids and proteins. We demonstrated the presence of an adaptation process to environmental heme in GAS and revealed a comprehensive transcriptome response to excess heme exposure. Finally, our findings also implicate a new gene cluster, *pefRCD*, in GAS heme sensing and tolerance.

We found that heme tolerance in GAS depends on medium type, growth conditions and genetic background (Table 3). Differences in bacterial metabolism, aeration, and cell-to-cell contact may all affect the bacterial sensitivity to heme. In addition, the THY medium is prepared from brain and heart infusion and is likely to contain some heme. Growth in THYB therefore, may allow for bacterial adaptation, possibly contributing to the higher heme MIC values observed in this medium. A significant variation in heme tolerance was exhibited by different strains of GAS; with NZ131, a serotype M49 skin isolate (Simon and Ferretti, 1991; Mcshan et al., 2008), demonstrating the lowest heme tolerance, whereas the more invasive strains such as M1T1 MGAS5005 (Sumby et al., 2005) and other clinical isolates showing higher heme resistance. It is tempting to speculate that these variations in heme sensitivity might add to the inclination of different GAS strains to colonize certain sites and to their invasive potential.

The absence of significant damage to cytosolic proteins in GAS suggests that the membrane is the primary target of heme in this bacterium (Figures 1, 2). This observation is in accordance with findings made in *S. aureus* (Wakeman et al., 2012) and is likely to result from heme accumulation in the cell envelope. The negative regulation of the heme uptake machinery works to limit the amount of heme that can reach the GAS cytoplasm (Bates et al., 2003; Montanez et al., 2005; Toukoki et al., 2010). In addition to active import, heme can diffuse through biological membranes, albeit with slow diffusion rates that can lead to accumulation in this compartment. Indeed it is well established that heme surplus tends to accumulate in the membranes of the eukaryotic cells, where it can be transformed into highly reactive species (Krishnamurthy et al., 2007; Chiabrando et al., 2014). There is less information regarding heme localization in bacteria; however, studies in *S. aureus* suggest a similar buildup in the cell envelope (Skaar et al., 2004). In addition to the preferential localization of heme outside of the cytoplasm, the reducing environment and the detox mechanism induced by heme (e.g., heat shock and redox factors) could mitigate some of the oxidative effects of the heme in this compartment. In this study we only probed the impact of heme at 4 μM, a concentration that is below the MIC level in early exponential phase, and for 90 min post treatment. Further investigations are needed to determine if heme at higher concentrations or after longer exposure time is affecting other cellular compartments.

The extended lag period that is observed when naïve GAS cells are introduced into medium containing heme and the absence of the growth delay in cells that were pre-exposed to low heme levels (Figure 3) provide strong evidence for the presence of heme sensing and coping mechanisms in GAS. Indeed, microarray analysis indicated that heme triggers comprehensive changes in GAS transcript levels (Table S1 and Figure 4A). The shift in the expression of regulatory proteins such as *spxA2, ihk/irr, ctsR*, and *hrcA* and their downstream-regulated genes, suggests that heme leads to significant redox, oxidative, and protein damage (Figure 4B). Spx is an RNA polymerase binding protein with CXXC redox-sensing center that acts to restore thiol balance by controlling redox enzymes and antioxidants formation (Nakano et al., 2003). The *ihk/irr* TCS regulates oxidative stress genes that protect GAS from killing by phagocytic cells and hydrogen peroxide (Voyich et al., 2004; Han et al., 2012). The induction of this regulatory system is consistent with ROS formation and cell envelope damage following heme exposure. The master regulators CtsR and HrcA, originally classified as class III and class I heat shock gene regulators, respectively, have been associated with a wide variety of stresses such as temperature shifts, antibiotics, carbonyl, electrophiles, etc. (Narberhaus, 1999; Elsholz et al., 2011). Here we show their association with heme stress and the elevated expression of chaperones and *clp* proteases further corroborates our hypothesis of heme-induced protein unfolding and findings of protein oxidation.

Heme treatment also resulted in significant inhibition of the genes encoding lactate oxidase (*lctO*) and of a V-type ATP synthases system (*ntpKCFABD*). LctO reaction leads to hydrogen peroxide production that can exacerbate the damage induced by heme (Seki et al., 2004; Kietzman and Caparon, 2010). The down regulation of the proton pump may serve to increase the reducing power at the cell surface. Further selective down regulation of genes such as *citDEFX* and up-regulation of *fruRBA*, which are involved in import and utilization of citrate and fructose, respectively, could indicate a metabolic state that is more conducive to detoxification.

Le Breton et al. recently used a *mariner*-based transposon library to screen for genes that are important for GAS survival in human blood (Le Breton et al., 2013). Comparing the heme stimulon identified in this study to the findings of Le Breton et al. revealed significant overlap, including *clpE* and *L, fhs.2, fruA, citF, trxS, lacZ*, and the *has* operon. This comparison suggests that heme, directly or indirectly, act as a signal that mediates adaptation *in vivo* and that the management of heme toxicity is relevant to GAS pathogenesis, in particular during spread through the blood.

The heme stimulon in GAS also includes three genes (*spy_0195-7)* that share high homology to the GBS *pefRCD* genes. In GBS these genes code for a heme responsive repressor (PefR) from the MarR-like family (Wilkinson and Grove, 2006) and to an ABC heme exporter (PefCD) (Fernandez et al., 2010). While the presence and genomic arrangements of *pefRCD* are highly conserved among different GAS strains (Figures 5, 6), we failed to identify homologs to *pefAB* (and *hrtAB*) that also participate in heme tolerance in GBS and other Gram-positive bacteria. The high degree of sequence similarity and the temporal increase in expression in response to heme stress (Figure 6B) suggest that the 3-gene locus encodes for a true homolog of the *pef* operon. This hypothesis is further supported by the identification of a putative PefR heme-binding box that is highly comparable to that of PefR from GBS (Figure 5A) (Fernandez et al., 2010) and by our heme and PPIX reconstitution experiments (Figures 7, 8). The ability of PefR to coordinate two heme molecules and curve fitting data demonstrating positive Hill's coefficient of cooperativity most likely suggest that the binding of heme at one binding site increases the affinity of heme binding at another site (Yifrach, 2004; Goutelle et al., 2008). Further experiments are underway to determine if and how heme and PPIX are modulating PefR binding to DNA and to establish the role of this 3-gene cluster in heme and protoporphyrin tolerance in GAS.

To the best of our knowledge our work is: (1) the first effort to characterize GAS mechanisms for heme toxicity and tolerance; (2) shows that environmental heme constitutes a significant redox stress in GAS with damaging effects on membrane lipids and proteins; (3) provides evidence for heme sensing and adaptation that involves global transcriptome shifts; and (4) links heme stress to several key regulators and TCS systems of this important human pathogen.

### Conflict of interest statement

The authors declare that the research was conducted in the absence of any commercial or financial relationships that could be construed as a potential conflict of interest.
