# Supplementary material for: The crimson conundrum: heme toxicity and tolerance in GAS
Source: Front Cell Infect Microbiol. 2014 Nov 5;4:159. doi: 10.3389/fcimb.2014.00159 (PMC4220732; doi:10.3389/fcimb.2014.00159)
Supplement: Supplementary file 1 [file DataSheet1.DOCX]

***Supplementary Material***

**The Crimson Conundrum: Heme Toxicity and Tolerance in GAS**

Ankita J Sachla^1^, Yoann Le Breton^2^, Fahmina Akhter^1^, Kevin S. McIver^2^ and Zehava Eichenbaum^1^

^1^ Department of Biology, College of Arts and Sciences, Georgia State University, Atlanta, GA 30302-4010, USA

^2^ Department of Cell Biology & Molecular Genetics and Maryland Pathogen Research Institute, University of Maryland, College Park, Maryland, 20742, USA

*** Correspondence:** Zehava Eichenbaum, Biology Department, College of Arts and Sciences, Georgia State University, P.O. Box 4010, Atlanta GA 30302-4010. Telephone: 404 413 5401. Fax: 404 413 5301. Email: [zeichen@gsu.edu](mailto:zeichen@gsu.edu)

## Supplementary Tables

**Supplementary** **Table S1. The *S. pyogenes* MGAS5005 Heme stimulon.**

|  | **Locus*^a^*** | **Gene^b^** | **Expression*^c^*** | **Putative function*^d^*** |
| --- | --- | --- | --- | --- |
| *Activated in the presence of Heme* | | | |  |
|  | Spy0013 | *ftsH* | 2.182 ± 0.033 | Cell division protein - COG0465 [O] |
|  | Spy0031 | *purK* | 2.01 ± 0.095 | Phosphoribosylaminoimidazole carboxylase ATPase subunit - COG0026 [F] |
|  | Spy0157 | *opuAA* | 2.638 ± 0.162 | GB transport ATP-binding protein - COG4175 [E] |
|  | Spy0158 | *opuABC* | 2.127 ± 0.147 | GB-binding protein/GB transport system permease protein - COG2113/4176 [E] |
|  | Spy0186 |  | 2.552 ± 0.086 | Transcriptional regulator - COG1846 [K] |
|  | Spy0195 | *pefR* | 2.778 ± 0.101* | Transcriptional regulator, MarR family - COG1846 [K] |
|  | Spy0196 | *pefC* | 2.001 ± 0.104 | MDR ABC transporter ATP-binding and permease protein - COG1132 [V] |
|  | Spy0197 | *pefD* | 2.112 ± 0.086 | MDR ABC transporter ATP-binding and permease protein - COG1132 [V] |
|  | Spy0275 |  | 2.673 ± 0.112 | Serine/threonine sodium symporter - COG3633 [E] |
|  | Spy0300 |  | 3.594 ± 0.091 | Hydrolase, HAD superfamily - COG1418 [R] |
|  | Spy0301 |  | 4.24 ± 0.103 | Integral membrane protein - COG0670 [R] |
|  | Spy0328 | *clpP* | 2.191 ± 0.061 | ATP-dependent Clp protease proteolytic subunit - COG0740 [O] |
|  | Spy0329 |  | 2.036 ± 0.127 | Hypothetical cytosolic protein |
|  | Spy0382 | *msrA.2* | 2.202 ± 0.108 | Peptide methionine sulfoxide reductase - COG0225 [O] |
|  | Spy0383 |  | 2.539 ± 0.109 | D-alanyl-D-alanine carboxypeptidase |
|  | Spy0385 |  | 2.609 ± 0.088 | 67 kDa Myosin-crossreactive antigen - COG0493 [E] |
|  | Spy0423 | *pepQ* | 2.162 ± 0.074 | Xaa-Pro dipeptidase - COG0006 [E] |
|  | Spy0425 |  | 2.235 ± 0.07 | Glycosyltransferase - COG0438 [M] |
|  | Spy0456 |  | 2.519 ± 0.122 | Plasmid stabilization system antitoxin protein - COG3077 [L] |
|  | Spy0457 |  | 2.185 ± 0.101 | Plasmid stabilization system protein - COG2026 [J/D] |
|  | Spy0474 | *licT* | 2.406 ± 0.099 | Transcription antiterminator, BglG family - COG3711 [K] |
|  | Spy0477 |  | 2.195 ± 0.061 | Hypothetical membrane spanning protein - COG3689 [S] |
|  | Spy0479 |  | 2.804 ± 0.078 | Hypothetical membrane spanning protein - COG0785 [O] |
|  | Spy0483 |  | 4.014 ± 0.105 | Stress-responsive transcriptional regulator - COG1983 [K/T] |
|  | Spy0509 | *tpi* | 3.168 ± 0.075 | Triosephosphate isomerase - COG0149 [G] |
|  | Spy0534 |  | 2.219 ± 0.102 | (R,R)-butanediol dehydrogenase/acetoin dehydrogenase - COG1028 [I/Q/R] |
|  | Spy0660 | *fruR* | 3.791 ± 0.165 | Fructose repressor - COG1349 [K/G] |
|  | Spy0661 | *fruB* | 4.285 ± 0.163 | 1-phosphofructokinase - COG1105 [G] |
|  | Spy0662 | *fruA* | 2.774 ± 0.153 | PTS system, fructose-specific IIABC component - COG1299/1445/1762 [G/T] |
|  | Spy0677 | *fms* | 2.111 ± 0.073 | Peptide deformylase - COG0242 [J] |
|  | Spy0694 | *clpL* | 3.928 ± 0.141 | Putative ATP-dependent Clp proteinase (ATP-binding subunit) - COG0542 [O] |
|  | Spy0743 |  | 3.181 ± 0.139 | ABC transporter substrate-binding protein - COG2984 [R] |
|  | Spy0744 |  | 2.488 ± 0.113 | Hypothetical protein |
|  | Spy0745 |  | 2.051 ± 0.147 | ABC transporter permease protein - COG4120 [R] |
|  | Spy0877 |  | 2.511 ± 0.082 | Hypothetical protein |
|  | Spy0878 |  | 2.527 ± 0.104 | Hypothetical protein |
|  | Spy0880 | *hlyIII* | 2.092 ± 0.123 | Putative hemolysin III - COG1272 [R] |
|  | Spy0910 | *citC* | 2.009 ± 0.058 | (Citrate [pro-3S]-lyase)-ligase - COG3053 [C] |
|  | Spy0924 |  | 2.105 ± 0.131 | Transcriptional regulator, GntR family |
|  | Spy0947 | *ciaH* | 2.353 ± 0.055 | TCS histidine kinase - COG1167 [K/E] |
|  | Spy0948 | *ciaR* | 2.094 ± 0.057 | TCS response regulator - COG074 [T/K] |
|  | Spy0982 |  | 2.188 ± 0.089 | Histidine-binding protein - COG0834 [E/T] |
|  | Spy1137 | *coiA* | 2.296 ± 0.12 | Putative competence protein/transcription factor |
|  | Spy1138 | *rsuA* | 2.823 ±0.136 | Ribosomal small subunit pseudouridine synthase A - COG1187 [J] |
|  | Spy1139 | *nagB* | 3.114 ± 0.132 | Glucosamine-6-phosphate isomerase - COG0363 [G] |
|  | Spy1203 |  | 2.074 ± 0.027 | Phage protein |
|  | Spy1222 | *int.2* | 1.94 ± 0.143 | Phi5005.2 integrase - COG0582 [L] |
|  | Spy1240 | *clpE* | 5.047 ± 0.174 | ATP-dependent clp protease ATP-binding subunit - COG0542 [O] |
|  | Spy1345 | *atoD.1* | 2.115 ± 0.122 | Acetate CoA-transferase alpha subunit - COG1788 [I] |
|  | Spy1406 | *copY* | 2.109 ± 0.102 | CopAB ATPases metal-fist type repressor - COG3682 [K] |
|  | Spy1477 |  | 2.34 ± 0.117 | Guanine-hypoxanthine permease - COG2252 [R] |
|  | Spy1497 | *dnaJ* | 3.327 ± 0.103 | Chaperone protein - COG0484 [O] |
|  | Spy1498 | *dnaK* | 4.58 ± 0.09 | Chaperone protein - COG0443 [O] |
|  | Spy1499 | *grpE* | 3.876 ± 0.112 | Hypothetical protein - COG0576 [O] |
|  | Spy1500 | *hrcA* | 4.081 ± 0.142 | Heat-inducible transcription repressor - COG1420 [K] |
|  | Spy1559 | *trx* | 2.491 ± 0.125 | Thioredoxin - COG0526 [O/C] |
|  | Spy1633 | *lacE* | 2.127 ± 0.066 | PTS system, lactose-specific IIBC component - COG1455 [G] |
|  | Spy1669 | *def* | 1.991 ± 0.091 | Peptide deformylase - COG0242 [J] |
|  | Spy1670 |  | 2.936 ± 0.101 | Oxidoreductase - COG0431 [R] |
|  | Spy1709 |  | 2.961 ± 0.17 | Hypothetical protein - COG3237 [S] |
|  | Spy1722 |  | 2.448 ± 0.082 | Hypothetical protein (Mga-associated) - COG0477 [G/E/P/R ] |
|  | Spy1723 | *isp* | 2.585 ± 0.131 | Immunogenic secreted protein - COG0810 [M] |
|  | Spy1724 | *ihk* | 2.554 ± 0.136 | TCS histidine kinase - COG0642 [T] |
|  | Spy1725 | *irr* | 2.719 ± 0.164 | TCS response regulator - COG0745 [T/K] |
|  | Spy1727 |  | 3.699 ± 0.118 | ABC transporter ATP-binding protein - COG1136 [V] |
|  | Spy1728 |  | 2.458 ± 0.127 | Periplasmic component of efflux system - COG0845 [M] |
|  | Spy1729 |  | 3.357 ± 0.148 | Hypothetical protein |
|  | Spy1730 |  | 2.691 ± 0.078 | Hypothetical protein |
|  | Spy1732 | *prsA* | 8.63 ± 0.043 | Protein export protein PrsA precursor - COG0760 [O] |
|  | Spy1753 | *pbp2A* | 2.173 ± 0.036 | Multimodular transpeptidase-transglycosylase - COG074 [M] |
|  | Spy1761 | *groEL* | 8.351 ± 0.134 | 60 kDa chaperonin - COG0459 [O] |
|  | Spy1762 | *groES* | 7.951 ± 0.134 | 10 kDa chaperonin - COG0234 [O] |
|  | Spy1763 | *clpC* | 2.576 ± 0.075 | Negative regulator of genetic competence - COG0542 [O] |
|  | Spy1764 | *ctsR* | 2.567 ± 0.124 | Transcriptional regulator |
|  | Spy1798 | *spxA2* | 5.238 ± 0.093 | suppressor of clpP & clpX, spxA2 allele - COG1393 [P] |
|  | Spy1853 | *hasC* | 2.204 ± 0.078 | UTP--glucose-1-phosphate uridylyltransferase - COG1210 [M] |
|  | Spy1865 | *htrA* | 3.539 ± 0.12 | Protease Do - COG0265 [O] |
| *Repressed in the presence of Heme* | | | |  |
|  | Spy0015 |  | 0.43 ± 0.158 | Hypothetical protein |
|  | Spy0040 | *adhA* | 0.368 ± 0.112 | Alcohol dehydrogenase - COG1064 [R] |
|  | Spy0117 |  | 0.333 ± 0.088 | Transcriptional regulator, LysR family - COG0583 [K] |
|  | Spy0118 |  | 0.374 ± 0.09 | Transcriptional regulator, LysR family - COG0583 [K] |
|  | Spy0127 | *ntpK* | 0.246 ± 0.275 | V-type sodium ATP synthase subunit K - COG0636 [C] |
|  | Spy0129 | *ntpC* | 0.219 ± 0.274 | V-type ATP synthase subunit C - COG1527 [C] |
|  | Spy0130 | *ntpF* | 0.219 ± 0.258 | V-type sodium ATP synthase subunit F - COG1436 [C] |
|  | Spy0131 | *ntpA* | 0.118 ± 0.306 | V-type sodium ATP synthase subunit A - COG1155 [C] |
|  | Spy0132 | *ntpB* | 0.092 ± 0.35 | V-type sodium ATP synthase subunit B - COG1156 [C] |
|  | Spy0133 | *ntpD* | 0.138 ± 0.328 | V-type sodium ATP synthase subunit D - COG1394 [C] |
|  | Spy0340 | *lctO* | 0.249 ± 0.124 | L-lactate oxidase - COG1304 [C] |
|  | Spy0361 |  | 0.458 ± 0.107 | Phosphoglycerate transporter protein - COG2271 [G] |
|  | Spy0562 | *sagA* | 0.31 ± 0.19 | Streptolysin S biosynthesis protein |
|  | Spy0563 | *sagB* | 0.325 ± 0.124 | Streptolysin S biosynthesis protein - COG0778 [C] |
|  | Spy0564 | *sagC* | 0.334 ± 0.116 | Streptolysin S biosynthesis protein |
|  | Spy0565 | *sagD* | 0.286 ± 0.129 | Streptolysin S biosynthesis protein |
|  | Spy0566 | *sagE* | 0.329 ± 0.105 | Streptolysin S putative self-immunity protein - COG1266 [R] |
|  | Spy0567 | *sagF* | 0.273 ± 0.135 | Streptolysin S biosynthesis protein - COG0477 [G/E/P/R] |
|  | Spy0568 | *sagG* | 0.282 ± 0.127 | Streptolysin S export ATP-binding protein - COG1131 [V] |
|  | Spy0569 | *sagH* | 0.304 ± 0.146 | Streptolysin S export transmembrane protein - COG0842 [V] |
|  | Spy0570 | *sagI* | 0.354 ± 0.113 | Streptolysin S export transmembrane protein - COG0842 [V] |
|  | Spy0571 |  | 0.312 ± 0.086 | Endonuclease/exonuclease/phosphatase family protein - COG2374 [R] |
|  | Spy0780 | *ptsA* | 0.069 ± 0.312 | PTS system, mannose/fructose family IIA component - COG2893 [G] |
|  | Spy0781 | *ptsB* | 0.073 ± 0.298 | PTS system, mannose/fructose family IIB component - COG3444 [G] |
|  | Spy0782 | *ptsC* | 0.093 ± 0.262 | PTS system, mannose/fructose family IIC component - COG3715 [G] |
|  | Spy0783 | *ptsD* | 0.369 ± 0.202 | PTS system, mannose/fructose family IID component - COG3716 [G] |
|  | Spy0784 | *spt5S* | 0.467 ± 0.112 | TCS histidine kinase - COG2972 [T] |
|  | Spy0830 | *dpiA* | 0.419 ± 0.129 | Transcriptional regulatory protein - COG4565 [K/T] |
|  | Spy0901 |  | 0.273 ± 0.174 | Hypothetical protein |
|  | Spy0905 | *citD* | 0.306 ± 0.183 | Citrate lyase acyl carrier protein - COG3052 [C] |
|  | Spy0906 | *citE* | 0.291 ± 0.169 | Citrate lyase beta chain/citryl-CoA lyase subunit - COG2301 [G] |
|  | Spy0907 | *citF* | 0.33 ± 0.129 | Citrate lyase alpha chain/citrate CoA-transferase - COG3051 [C] |
|  | Spy0908 | *citX* | 0.315 ± 0.107 | Apo-citrate lyase phosphoribosyl-dephospho-CoA transferase - COG3697 [H/I] |
|  | Spy0929 |  | 0.479 ± 0.089 | SIR2 family protein - COG0846 [K/E] |
|  | Spy0930 |  | 0.428 ± 0.103 | ATPase associated with chromosome architecture/replication - COG2110 [R] |
|  | Spy0931 |  | 0.411 ± 0.1 | Glycine cleavage system H protein - COG0509 [E] |
|  | Spy0932 |  | 0.416 ± 0.102 | Luciferase-like monooxygenase - COG2141 [C] |
|  | Spy0933 |  | 0.428 ± 0.085 | Probable NADH-dependent flavin oxidoreductase - COG1902 [C] |
|  | Spy0940 |  | 0.48 ± 0.059 | Nucleoside transport system permease protein - COG4603 [R] |
|  | Spy0941 |  | 0.417 ± 0.067 | Nucleoside transport ATP-binding protein - COG3845 [R] |
|  | Spy0942 |  | 0.459 ± 0.079 | Nucleoside-binding protein - COG1744 [R] |
|  | Spy0944 |  | 0.443 ± 0.074 | 16S rRNA m(2)G 1207 methyltransferase - COG2813 [J] |
|  | Spy0951 | *pstB* | 0.491 ± 0.046 | Phosphate transport ATP-binding protein - COG1117 [P] |
|  | Spy1058 | *malE* | 0.373 ± 0.135 | Maltose/maltodextrin-binding protein - COG2182 [G] |
|  | Spy1076 | *glnH* | 0.422 ± 0.047 | Transporter - COG0765 [E/T ] |
|  | Spy1084 |  | 0.468 ± 0.118 | Outer surface protein - COG3589 [S] |
|  | Spy1161 |  | 0.346 ± 0.12 | Formate transporter - COG2116 [P] |
|  | Spy1270 | *arcC* | 0.283 ± 0.152 | Carbamate kinase - COG0549 [E] |
|  | Spy1271 |  | 0.278 ± 0.104 | Xaa-His dipeptidase - COG0624 [E] |
|  | Spy1272 |  | 0.315 ± 0.125 | Arginine/ornithine antiporter - COG1288 [S] |
|  | Spy1273 | *arcB* | 0.402 ± 0.091 | Ornithine carbamoyltransferase - COG0078 [E] |
|  | Spy1286 |  | 0.483 ± 0.056 | DNA polymerase - COG1518 [L] |
|  | Spy1303 | *aroE* | 0.461 ± 0.149 | Shikimate 5-dehydrogenase - COG0169 [E] |
|  | Spy1304 | *lacZ* | 0.306 ± 0.174 | Beta-galactosidase - COG3250 [G] |
|  | Spy1305 | *trxR* | 0.243 ± 0.158 | TCS response regulator - COG4753 [T] |
|  | Spy1306 | *trxS* | 0.345 ± 0.162 | TCS histidine kinase - COG2972 [T] |
|  | Spy1307 |  | 0.22 ± 0.145 | Hypothetical membrane spanning protein |
|  | Spy1308 |  | 0.168 ± 0.201* | Sugar-binding protein - COG1653 [G] |
|  | Spy1309 |  | 0.137 ± 0.18 | Sugar transport system permease protein - COG0395 [G] |
|  | Spy1310 |  | 0.184 ± 0.146 | Sugar transport system permease protein - COG4209 [G] |
|  | Spy1315 |  | 0.454 ± 0.053 | Transcriptional regulator, GntR family - COG1609 [K] |
|  | Spy1316 |  | 0.459 ± 0.082 | Hypothetical protein - COG3538 [S] |
|  | Spy1376 |  | 0.234 ± 0.182 | Transaldolase - COG0176 [G] |
|  | Spy1377 |  | 0.239 ± 0.136 | *trans*-acting positive regulator |
|  | Spy1378 |  | 0.322 ± 0.099 | NADH peroxidase - COG0446 [R] |
|  | Spy1379 | *glpF* | 0.209 ± 0.184 | Glycerol uptake facilitator protein - COG0580 [G] |
|  | Spy1380 | *glpO* | 0.181 ± 0.133 | Alpha-glycerophosphate oxidase - COG0578 [C] |
|  | Spy1381 | *glpK* | 0.085 ± 0.255 | Glycerol kinase - COG0554 [C] |
|  | Spy1539 | *scrK* | 0.32 ± 0.088 | Fructokinase - COG1940 [K] |
|  | Spy1540 | *endoS* | 0.176 ± 0.105 | Endo-beta-N-acetylglucosaminidase F2 precursor |
|  | Spy1596 | *glnA* | 0.409 ± 0.112 | Glutamine synthetase - COG0174 [E] |
|  | Spy1597 | *glnR* | 0.389 ± 0.078 | Transcriptional regulator, MerR family - COG0789 [K] |
|  | Spy1682 | *msmK* | 0.392 ± 0.131 | Multiple sugar transport ATP-binding protein - COG3839 [G] |
|  | Spy1758 |  | 0.5 ± 0.099 | Probable dipeptidase B - COG4690 [E] |
|  | Spy1771 | *hutU* | 0.242 ± 0.146 | Urocanate hydratase - COG2987 [E] |
|  | Spy1772 |  | 0.22 ± 0.192 | Glutamate formiminotransferase - COG3643 [E] |
|  | Spy1773 |  | 0.16 ± 0.178 | Formiminotetrahydrofolate cyclodeaminase - COG3404 [E] |
|  | Spy1774 | *fhs.2* | 0.136 ± 0.138 | Formate--tetrahydrofolate ligase - COG2759 [F] |
|  | Spy1775 |  | 0.15 ± 0.16 | Hypothetical cytosolic protein - COG3758 [S] |
|  | Spy1776 |  | 0.154 ± 0.182 | Amino acid permease - COG0531 [E] |

*^a^*Spy numbers from MGAS5005 genome.

*^b^*When available, gene name is provided.

*^c^*Array mean ± standard error. Numbers with an asterisk represent the average of array means obtained from 2 or more distinct probes for the same locus.

*^d^*Putative function using nomenclature from the J. Craig Venter Institute (JCVI) CMR database. ABC, ATP-binding cassette; CoA, coenzyme A; GB, Glycine betaine; MDR, multiple drug resistance; PTS, PEP-dependant phosphotransferase system; TCS, two-component system. Further information relative to the gene-encoded protein is provided using the NCBI Clusters of Orthologous Groups (COG) database. Function categories are given using the letter-based nomenclature from the COG database. Information storage and processing: J, translation, ribosomal struture and biogenesis; A, RNA processing and modification; K, transcription; L, replication, recombinaison and repair; B, chromatin structure and dynamics. Cellular processes and signaling: D, cell cycle control, cell division, chromosome partitioning; Y, nuclear structure; V, defense mechanisms; T, signal transduction mechanisms; M, cell wall/membrane/envelope biogenesis; N, cell motility; Z, cytoskeleton; W, extracellular structures; U, intracellular trafficking, secretion, and vesicular transport; O, posttranslational modification, protein turnover, chaperones. Metabolism: C, energy production and conversion; G, carbohydrate transport and metabolism; E, amino acid transport and metabolism; F, nucleotide transport and metabolism; H, coenzyme transport and metabolism; I, lipid transport and metabolism; P, inorganic ion transport and metabolism; Q, secondary metabolites biosynthesis, transport and catabolism. Poorly characterized: R, general function prediction only; S, unknown function.

## Supplementary Figures


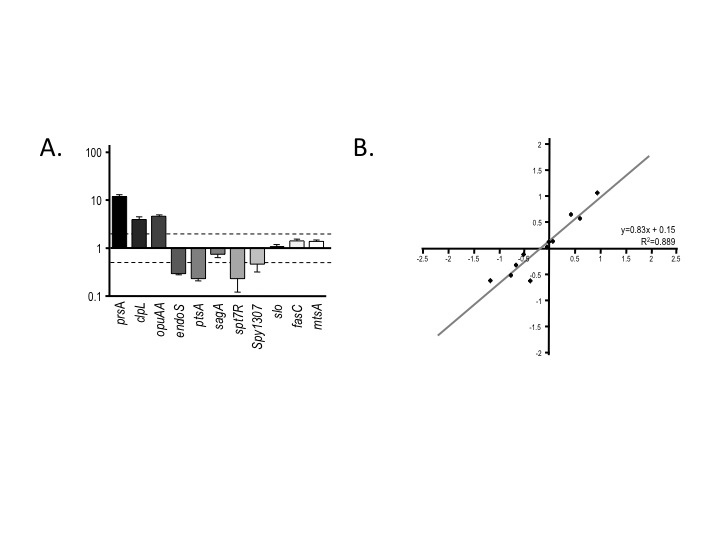


Supplementary Figure S1. Microarray data validation. (A) A set of 11 genes representing the range of possible changes, i.e. increase (*prsA*, *clpL*, *opuAA*), decrease (*endoS*, *ptsA*, *sagA*, *spt7R*) and no effect (*slo*, *fasC*, *mtsA*) were selected and their expression analyzed by real-time RT-PCR. (B) Plotting the log value of the array on the x-axis against the log value of the real-time RT-PCR on the y-axis revealed strong correlation (calculated R^2^ = 0.892) between the two datasets.
